# Photochemically-Mediated Inflammation and Cross-Presentation of *Mycobacterium bovis* BCG Proteins Stimulates Strong CD4 and CD8 T-Cell Responses in Mice

**DOI:** 10.3389/fimmu.2022.815609

**Published:** 2022-01-31

**Authors:** Ying Waeckerle-Men, Zuzanna K. Kotkowska, Géraldine Bono, Agathe Duda, Isabel Kolm, Eleni M. Varypataki, Beat Amstutz, Michael Meuli, Anders Høgset, Thomas M. Kündig, Cornelia Halin, Peter Sander, Pål Johansen

**Affiliations:** ^1^ Department of Dermatology, University of Zurich and University Hospital Zurich, Zurich, Switzerland; ^2^ Institute of Medical Microbiology, University of Zurich, Zurich, Switzerland; ^3^ PCI Biotech Holding ASA, Oslo, Norway; ^4^ Institute of Pharmaceutical Sciences, ETH Zurich, Zurich, Switzerland; ^5^ National Center for Mycobacteria, University of Zurich, Zurich, Switzerland

**Keywords:** PCI facilitates BCG vaccination, photochemical internalization, T cells, tuberculosis, vaccine, cross-presentation

## Abstract

Conventional vaccines are very efficient in the prevention of bacterial infections caused by extracellular pathogens due to effective stimulation of pathogen-specific antibodies. In contrast, considering that intracellular surveillance by antibodies is not possible, they are typically less effective in preventing or treating infections caused by intracellular pathogens such as *Mycobacterium tuberculosis*. The objective of the current study was to use so-called photochemical internalization (PCI) to deliver a live bacterial vaccine to the cytosol of antigen-presenting cells (APCs) for the purpose of stimulating major histocompatibility complex (MHC) I-restricted CD8 T-cell responses. For this purpose, *Mycobacterium bovis* BCG (BCG) was combined with the photosensitiser tetraphenyl chlorine disulfonate (TPCS2a) and injected intradermally into mice. TPCS2a was then activated by illumination of the injection site with light of defined energy. Antigen-specific CD4 and CD8 T-cell responses were monitored in blood, spleen, and lymph nodes at different time points thereafter using flow cytometry, ELISA and ELISPOT. Finally, APCs were infected and PCI-treated *in vitro* for analysis of their activation of T cells *in vitro* or *in vivo* after autologous vaccination of mice. Combination of BCG with PCI induced stronger BCG-specific CD4 and CD8 T-cell responses than treatment with BCG only or with BCG and TPCS2a without light. The overall T-cell responses were multifunctional as characterized by the production of IFN-γ, TNF-α, IL-2 and IL-17. Importantly, PCI induced cross-presentation of BCG proteins for stimulation of antigen-specific CD8 T-cells that were particularly producing IFN-γ and TNF-α. PCI further facilitated antigen presentation by causing up-regulation of MHC and co-stimulatory proteins on the surface of APCs as well as their production of TNF-α and IL-1β *in vivo*. Furthermore, PCI-based vaccination also caused local inflammation at the site of vaccination, showing strong infiltration of immune cells, which could contribute to the stimulation of antigen-specific immune responses. This study is the first to demonstrate that a live microbial vaccine can be combined with a photochemical compound and light for cross presentation of antigens to CD8 T cells. Moreover, the results revealed that PCI treatment strongly improved the immunogenicity of *M. bovis* BCG.

## Introduction

The only approved vaccine against *Mycobacterial tuberculosis* (*Mtb*) is *Mycobacterium bovis* Bacille Calmette–Guérin (BCG), which has variable protective efficacy (1–4). WHO recommends BCG in HIV-uninfected infants and juveniles as it provides protection against severe extrapulmonary tuberculosis forms, e.g. miliary and meningeal tuberculosis. However, after almost one century of *M. bovis* BCG vaccination, tuberculosis (TB) still causes more than a million of fatalities each year. The conventional BCG vaccine has shown poor protection efficacy, especially in pulmonary TB in adults (5–7). In addition, BCG typically fails to protect immunocompromised or -suppressed persons (8, 9). Finally, due to the ever-growing problem of multidrug-resistant *Mtb*, complementary healthcare interventions including new and effective prophylactic and therapeutic TB vaccines are urgently required.

The reason of the suboptimal protective efficacy of the *M. bovis* BCG vaccine is still not clear. It has been hypothesized that the attenuation process of *M. bovis* BCG caused by the loss of type VII secretion system ESX1 and loss some important *Mtb*-associated antigens such as a 10-kDa culture filtrate protein (CFP-10) and a 6-kDa early-secreted antigenic target (ESAT-6) thereby compromising its immunogenicity (2, 10). Moreover, some homologues of *M. tuberculosis* virulence proteins still conserved in *M. bovis* BCG may inhibit the capacity of processing and presentation of mycobacterial antigens by antigen-presenting cells (APCs), including macrophages and dendritic cells (DCs), thus, shielding the pathogen from induction of immune responses (11, 12).

T-cell mediated immunity plays an important role in controlling TB infection (13–16). As intracellular bacteria, *M. bovis* BCG are phagocytosed by macrophages, and the mycobacterial antigens subsequently processed and presented *via* a MHC class II-restricted pathway, to favor the activation of the CD4 T cell arm of immunity. Such CD4 T-cell responses are associated with the production and secretion of T-helper type 1 (Th1) cytokines, which may have bactericidal effects by activation of nitric oxide (NO) production in *Mtb* infected cells (17–19). It has also been shown that both *Mtb* and *M. bovis* BCG can arrest phagosome maturation, which again will prevent antigen presentation and immunity (20–23). On the other hand, CD8 T cells are also essential in preventing TB through specific cytokines, e.g. TNF-α and IFN-γ, cytotoxic granules such as perforin and granzymes, and through expression of Fas ligand that binds Fas on infected cells and drives them into apoptosis (24–27).

Many alternatives to the *M. bovis* BCG vaccine are in pre- and clinical development (7, 11, 28–40). Most of these developments are aimed to improve the immunogenicity of *M. bovis* BCG, either by genetic modifications, by choosing effective antigen subsets from *Mtb*/BCG (41–46), by adding more improved adjuvants (47), or by using prime-boost strategies (48–50). Moreover, the route for administration may also be important for of the vaccine efficacy (26, 51). We have shown that direct intralymphatic inoculation of BCG into lymph nodes strongly improved T-cell responses in mice (52). Kaushal et al. have reported that the mucosal vaccination with an attenuated *Mtb* mutant induced strong bronchus-associated tissue (iBALT) as well as T-cell responses in macaques and protected TB (53). More recently, it has been demonstrated that intravenous BCG vaccination prevented TB in macaques, and the protection strongly correlated with CD8 and CD4 T-cell responses in the secondary lymphoid organs and in lung tissues, therefore, further implying the importance of an optimal T-cell function (26).

Photodynamic therapy (PDT) is widely used in dermatology for the treatment of infections and pre-cancerous skin lesions. Its principle involves the light activation of a photosensitizer (PS), which triggers a photochemical reaction including highly reactive oxygen species (ROS) that may cause disruption of cell membranes (54, 55). Based on the function of PDT, we and others have developed a novel immunization strategy by combining antigens with photosensitizer [reviewed in (56)]. Its aim is to trigger a photochemical internalization (PCI) of the antigen into the cytosol of APCs (57–59). The proposed mechanism of PCI is that after uptake, e.g. phago/endocytosis, of antigens and photosensitizer by APCs, subsequent light treatment invokes a ROS-mediated disruption of endosomal membranes and the release of endosomal content into cytosol. By this process, the antigens get access to MHC class I molecules and can be cross-presented to CD8 T cell. Previous studies demonstrated that such PCI-based vaccination enhanced and elicited high level of antigen-specific CD8 T-cell responses towards soluble (59–61) and particulate (62–64) protein antigens. It was also showed that PCI-mediated vaccination was also effective for preventing and treating cancer (65, 66). In the present study, we evaluated the feasibility of applying PCI to a live vaccine such as *M. bovis* BCG, with the objective of triggering CD8 T-cell responses with potential protection against TB. Antigen-specific T-cell immune responses were monitored after vaccination of mice, and the data suggest that PCI induces an adjuvant-like local inflammatory response and enables the cross presentation of *M. bovis* BCG proteins as it clearly improves specific CD8 and CD4 T-cell responses.

## Materials and Methods

### Mice

Wild type C57BL/6 and BALB/c female mice 6-8 weeks of age were purchased from Envigo (Horst, the Netherlands) or Janvier (Genest-Saint-Isle, France) and used age-matched after 1-3 weeks of acclimatization in the animal facility *Biologisches Zentrallabor* of the University Hospital of Zurich. Rag2-deficient transgenic OT-I mice (B6.129S6-Rag2*^tm1Fwa^* Tg(TcraTcrb)1100Mjb) that recognize the MHC class-I-restricted H-2K^b^ epitope SIINFEKL from ovalbumin (aa257-264_)_ were purchased from Taconic Europe (Ry, Denmark) and further bred in the animal facility. All animals were kept under specific pathogen free conditions, in individually ventilated cages, in groups of 4-5 mice, at 21°C, and with a 12h-12h light-dark cycle. Experiments took place during daytime and under laminar airflow in biosafety hoods. The animal experiments were reviewed by the local ethical review board, approved by the Veterinary authorities of canton Zurich (ZH 52/2016 and ZH 170/2019), and performed in accordance with Swiss animal law and regulations.

### Materials

Purified protein derivative (PPD; Tuberculin PPD), was bought from AJ Vaccines (Copenhagen, DK). Mouse H-2K^b^ MHC class I-restricted peptides IMYNYPAM (Mycobacterium tuberculosis TB10.4 4-11) (67) and SIINFEKL (Ovalbumin aa257-264) and peptide-MHC-pentamer complexes for flow cytometry were purchased from Proimmune (Oxford, UK). The photosensitizer tetraphenyl chlorine disulfonate (TPCS2a) (68) was provided by PCI Biotech (Oslo, NO). Phosphate-buffered saline (PBS), cell culture medium RPMI-1640, Fetal Calf Serum (FCS), L-Glutamine, and Normocin were bought from Sigma-Aldrich (Buchs, CH). Cytokine ELISA/ELISPOT kits and all fluorophore-labeled antibodies used for flow cytometry were purchased from eBioscience/Invitrogen (Thermo Fisher Scientific, San Diego, CA).

### Cells and BCG Bacteria Strains

Mouse macrophage cell line RAW264.7 (ATCC^®^TIB-71™, BALB/c derived H-2d) were purchased from ATCC (Manassas, VA). Cells were cultured in RPMI-1640 medium supplemented with 10% FCS, 2 mM L-glutamine and 0.1 mg/ml Normocin (RPMI-C medium).

Three different strains of *M. bovis* BCG were used in this study: (i) Pasteur BCG strain *M. bovis* BCG 1721, a streptomycin resistant derivative of *M. bovis* BCG Pasteur (69); (ii) Pasteur BCGΔ*zmp1* with depleted *zinc metalloprotease 1* (*zmp1*) gene (70); (iii) Denmark *M. bovis* BCG strain (DK BCG::OVA) that was genetically modified to express a mini gene comprised of the 19kDa lipoprotein (lpqH) with C-terminal fusion of the immune dominant H-2k^b^ mouse CD8 T-cell epitope SIINFEKL (71). All BCG strains were produced in house and grown in Middlebrook 7H9 broth supplemented with 0.05% Tween-80 and Middlebrook oleic acid-albumin-dextrose-catalase obtained from BD Biosciences (Becton Dickinson, Franklin Lakes, NJ).

### Intradermal BCG Vaccination and Photochemical Activation

One day prior to vaccination, C57BL/6 mice were shaved in the abdominal region to enable better light exposure of the skin. On the day of vaccination, 50 µg TPCS2a was mixed with the 1-4×10^6^ CFU *M. bovis* BCG in PBS, kept light protected, and used for injection within 60 min. A total volume of 100 µl of the vaccine was injected intradermally (*i.d.*) in the shaved left and right abdominal region (50 µl each) using 500 µl syringes with 29G needles. After 18 hours, the mice were anaesthetized with intraperitoneal injection of ketamine (25mg/kg body weight) and xylazine (4 mg/kg body weight) mixture in PBS. The sedated mice were then placed belly-down on the LumiSource^®^ (PCI Biotech) light source and illuminated with blue light (peak emission at 435 nm) at 4.86 J/cm^2^ (six minutes) as described previously (60).

To facilitate monitoring of MHC class I-restricted CD8 T-cell activation *in vivo*, a vaccination protocol including adoptive transfer of CD8 T cell was also used (60). In short, 1×10^6^ of naïve freshly prepared cells from lymph nodes (LNs) and spleen of transgenic OT-I mice were intravenously (*i.v.*) injected into the recipient C57BL/6 mice one day prior to vaccination with BCG : OVA and 50 µg TPCS2a. The injected skin areas were illuminated 18 hours later as above, while the head, thorax, and legs were protected from light using aluminum foil.

### Intravenous BCG Vaccination

In some experiments, and in order to compare the effect of PCI-based BCG vaccination with a reported effective BCG vaccination (26), C57BL/6 mice were vaccination with *M. bovis* BCG by *i.v.* injection. Briefly, mice were pre-warmed under the red warm lamp for 5 minutes, the local tail veins were disinfected with 70% ethanol, and 4×10^6^ CFU of *M. bovis* BCG in 100 µl PBS were injected into the tail vein using 300 µl syringes with 30G needles.

### Delayed-Type Hypersensitivity Test

A delayed-type hypersensitivity (DTH) reaction in BCG vaccinated mice was performed using mouse ear-swelling test as previously described (72). Briefly, two weeks after *i.d.* injection of BCG with or without concomitant PCI treatment, mice were challenged by an *i.d.* injection of 10 μl of a 1 mg/ml PPD solution in PBS into the pinna of the left ear. Before injection, as well as 24, 48, and 72 hours after the challenge, the DTH reaction was monitored by measuring the ear thickness using a spring-loaded Mitutoyo (Kawasaki, Japan) precision digital thickness gauge (provided by Brütsch-Rüegger Corp., Urdorf, CH). As a negative control, 10 μl PBS was injected into the right ear, and the ear swelling was calculated by subtracting right-ear values (neg. control) form left-ear values (PPD samples).

### Flow Cytometry

Intracellular cytokine analysis in spleen and LN cells was performed using the protocols provided by the manufacturer (BD Biosciences). The cells were suspended in RPMI-C medium and re-stimulated with 5 µg/ml PPD overnight, or with 1 µM SIINFEKL or IMYNYPAM peptide at 37°C, 5% CO_2_ for 6 hours; splenocytes were treated with Red Blood Cell Lysis Buffer (Sigma-Aldrich) before incubation. During the last 4 hours, 5 µg/ml Brefeldin A (Sigma-Aldrich) was added. The cells were then washed in cold PBS, fixed with Cytofix/Cytoperm™ solution, and permeabilized with Perm/Wash™ solution (BD Biosciences, San Diego, CA). The cells were incubated with anti-CD16/CD32 Ab (clone 93) for 15 minutes to block FcRs then stained with rat anti-mouse CD4 PE (GK1.5), CD8 PerCP-Cy5.5 (53-6.7), CD44-FITC (IM7), IFN-γ APC (XMG1.2), and TNF-α PE-Cy7 (MP6-XT22) for 45 minutes. All antibodies were from Invitrogen and used at 1:200 dilution. All steps were intercepted by washing in cold PBS, and the incubations were performed protected from light and on ice. The samples were acquired using a FACSCanto flow cytometer (BD Biosciences) and results were analyzed using FlowJo v.10 software (Tree Star).

To monitor the frequency of antigen-specific CD8 T cells SIINFEKL or IMYNYPAM, venous blood was collected and erythrocytes were lysed with Red Blood Cell Lysis Buffer (Sigma-Aldrich) before FcRs blocking with anti-CD16/CD32 Ab. The mouse peripheral blood mononuclear cells (PBMCs) were stained with PE-conjugated H-2K^b^/Pro5 peptide-specific pentamers (Proimmune) for 15 minutes 37°C and then with anti-CD8-PerCP-Cy5.5 and anti-CD44-FITC for 45 minutes.

### IFN-γ ELISPOT Assay

Antigen-specific IFN-γ production was analyzed by mouse IFN-γ ELISPOT assay according to the manufacturer’s protocol (eBioscience). Briefly, multiscreen 96-well PVDF plates (Millipore, Wohlen, Switzerland) were coated with 1 μg/ml anti-mouse IFN-γ antibody at 4°C overnight. The plates were then blocked with RPMI-C medium before freshly prepared splenocytes were seeded at 2×10^5^ cells per well) and re-stimulated with the antigens indicated (5 µg/ml of PPD, 1 µg/ml IMYNYPAM or SIINFEKL peptides) at 37°C, 5% CO_2_ and for 18 hours. ELISPOT plates were developed according to the manufacturer’s protocol and spots were read with AID EliSpot Reader System (Autoimmun Diagnostika, Strassberg, DE).

### Cytokine ELISA Analysis

ELISA was performed to analyze cytokine secretion in supernatants of splenocytes after *in vitro* antigen re-stimulation. Briefly, 5×10^5^ splenocytes were re-stimulated 24-72 hours in round-bottom 96-well plates with the antigens indicated (5 µg/ml of PPD, 0.1 µg/ml IMYNYPAM or SIINFEKL peptides, respectively) and cultured at 37° C, 5% CO_2_. Cell culture supernatants were collected at different time points and analyzed for secretion of IL-2, TNF-α (24 h), IFN-γ and IL-17A (72 h) using Ready-Set-GO^®^ ELISA Kits as described by the manufacturer (eBioscience).

### *Ex Vivo* Antigen Presentation Assay

An ex vivo antigen presentation assay was used to judge whether PCI affects the cross-presentation of *M. bovis* BCG to epitope-specific CD8 T cells. Briefly, C57BL/6 mice were vaccinated i.d. with 4×10^6^ CFU *M. bovis* BCG::OVA and treated with PCI as described above. Two days after vaccination, inguinal, axillary, and mesenteric LNs as well as spleens from vaccinated mice were harvested. Single-cell suspensions were prepared and used as APCs (1×10^5^ cells/well) in co-cultures with naïve SIINFEKL-specific CD8 T cells isolated from OT-I transgenic mice (2×10^4^ cell/well). The cultures in round-bottom 96-well plates were incubated at 37°C, 5% CO2 for 72 hours. The antigen presentation capacity was then determined by analyzing IFN-γ in the culture supernatants by ELISA (eBiosciences).

### *In Vitro* Infection of RAW264.7 Macrophages With BCG and PCI and Analysis of Inflammatory Responses

*M. bovis* BCG 1×10^7^ CFU of and photosensitizer TPCS2a (0.5 µg) were mixed in 1 ml of PBS and incubated at 37°C in dark to initiate binding of TPCS2a to BCG. Two hours later, BCG were washed thrice with PBS to remove free TPCS2a. The suspensions of BCG-TPCS2a or BCG was then added to RAW264.7 macrophages in RPMI-C medium and in 6 well plates (1×10^6^ BCG and 5×10^5^ cells per ml), and the infected cultures were incubated over night at 37°C and 5% CO_2_ under light protection. The RAW cells were then collected, washed thrice with PBS and illuminated (2.43 J/cm^2^; 3 minutes). One part of the infected RAW264.7 cells were used for testing macrophage activation *in vitro*, another part of the cells were used for intralymphatic injection (see below).

The activation of RAW264.7 macrophages was monitored by intracellular cytokine staining and flow cytometry as described above. Briefly, after 4 hours incubation with brefeldin A, cells were washed, fixed, and permeabilized. After anti-CD16/CD32 treatment, the cells were stained with rat anti-mouse CD11b-FITC (clone M1/70), Pro IL-1β-PE (NJTEN3) and TNF-α-PE-Cy7 (all Invitrogen) for 45 minutes. The surface expression of MHC-I, MHC-II and CD86 on BCG-PCI loaded macrophages were measured three days after the light treatment.

### Vaccination of Mice With BCG Infected and PCI Treated Macrophages

BCG and PCI-treated RAW264.7 macrophages were also used for vaccination of BALB/c mice by direct intralymphatic injection as described (73, 74). Briefly, the mice were anaesthetized with ketamine and xylazine, and a small skin incisions in the left and right inguinal region were made aseptically. Using 500 µl syringes with 29G needles, 5×10^5^ RAW264.7 cells in 10 µl was injected directly into each of the two lymph nodes. The skin incisions were closed using surgical thread. Six days later, mice were euthanized and the injected inguinal LNs were harvested. Antigen-specific T cells as wells as CD11b-positive myeloid cells were assessed for cytokine productions by flow cytometry as described above.

### Histological Examinations of Skin From BCG- and PCI-Treated Mice

C57BL/6 mice were injected intradermally in the belly region with BCG with or without TPCS2a as described above. After light treatment on day 1, skin samples were collected on days 2 and 8 post BCG administration, fixed in 4% formalin in PBS overnight, dehydrated, and embedded in paraffin. Sections of 3 μm were cut on a microtome and stained with hematoxylin and eosin (H&E). Additional sections were subjected to immunohistochemistry for identification of CD11b myeloid cells (monocytes, macrophages, neutrophils and granulocytes), CD11c DCs, CD68 macrophages, as well as CD4 and CD8 T cells (Sophistolab AG, Muttenz, Switzerland). The sections were evaluated by a board certified dermatopathologist, scanned on Aperio ScanScope CS, and the images prepared using Aperio ImageScope software v11.2.0.780. Inflammatory infiltrates in different regions were manually counted from the representative sections by two independent researchers, using 6-7 frames per section, and by using QuPath software (75).

### Statistical Analysis

**Figure 1:**
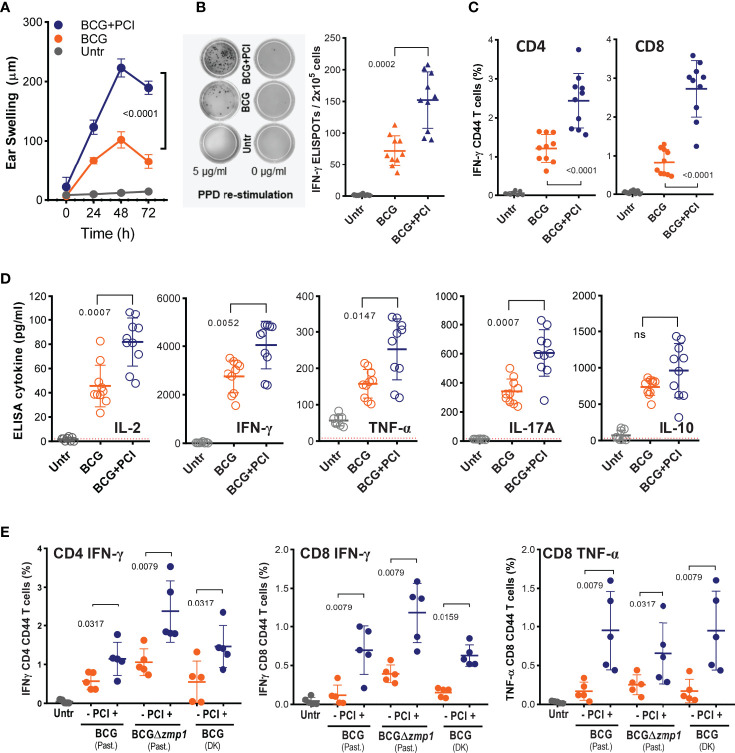
PCI-based BCG vaccination induced strong PPD-specific CD8 and CD4 and T-cell responses in mice. **(A-E)** Groups of 8-10 C57BL/6 mice were vaccinated *i.d.* in the belly with 4×10^6^ CFU of Pasteur BCG (BCG), with a mixture of BCG and 50 µg TPCS2a (BCG+PCI) or left untreated (Untr). After 18 h, the mice were light treated. **(A)** Two weeks later, mice were challenged *i.d.* with 10 µg of PPD injected in the ear skin. DTH-induced ear swelling was measured at 0, 24, 48, and 72 hours after the challenge. **(B, C)** PPD specific T-cell responses were assessed in splenocytes by IFN-γ ELISPOT assay **(B)** for total T cells or by flow cytometry **(C)**, gating on IFN-γ expression in CD4^+^CD44^+^ and CD8^+^CD44^+^ T cells. **(D)** Splenocytes from immunized mice were also re-stimulated *in vitro* with PPD and cytokine secretion measured by ELISA. Dotted lines indicate the detection limits. **(E)** Pasteur (Past) BCG, Pasteur BCGΔzmp1, and Denmark (DK) BCG, were used for vaccination ± PCI as above. Three weeks later, IFN-γ-producing and CD44-expressing CD4 and CD8 T cells were monitored by flow cytometry. Representative data are shown as means ± SD. For statistical analysis, two-way ANOVA test was applied to the DTH responses between BCG and BCG+PCI groups. To compare T-cell activation, 2-tailed Mann Whitney U-tests were performed. Experiments were repeated at least two times with comparable results. ns, not significant.

All experiments were repeated at least two times, and representative experiments are shown. Results are presented as individual dots/bars with mean ± standard deviation (SD), or as box plots with median and 5-95% whiskers. The statistical analysis was evaluated by GraphPad Prism 8 (GraphPad Software Inc., La Jolla, CA, USA). Typically, non-parametric two-tailed Mann-Whitney U test was used to compare two test groups, while non-parametric two-sided Kruskal-Wallis with Dunn’s multiple comparisons test was used to compare three or more test groups. Some data was analyzed by one-way ANOVA with Bonferroni’s multiple comparison test (**Figures 3B**, **6**) For dynamic data, a parametric two way ANOVA test was applied (**Figures 1A**, **5A**). The significance level was set to 95%.

## Results

### Application of PCI With *M. bovis* BCG Improved T-Cell Immunity

C57BL/6J mice were vaccinated *i.d*. with a mixture of *M. bovis* BCG and photosensitizer TPCS2a and treated with light after 18 hours. Two weeks after vaccination, a cutaneous DTH reaction to mycobacterial proteins (PPD) was determined as a measure for cellular immunity. PPD-specific ear swelling was observed 24-72 hours post challenge in all BCG-vaccinated mice (**Figure 1A**). At the peak of the DTH reaction (48 h), approx. 100 µm swelling was measured in BCG-vaccinated mice, while mice that also received PCI treatment showed a swelling of approx. 225 µm, suggesting that PCI increased the immunogenicity of BCG (p<0.0001 by 2-way ANOVA). No ear swelling was observed in non-vaccinated mice or after challenge with PBS. The mice were then euthanized and the splenocytes re-stimulated *in vitro* with PPD. ELISPOT revealed that the frequency of PPD-specific IFN-γ-producing T cells was increased after combined BCG vaccination and PCI treatment as compared to BCG vaccination alone (**Figure 1B**). Furthermore, flow cytometry revealed increased PPD-specific intracellular IFN-γ production after PCI-based BCG vaccination, for both of CD4 and CD8 T cells (**Figure 1C**). The representative gatings were shown in the **Supplementary Figure S1**. The phenotype of T-cell responses was further characterized by ELISA in cultures of splenocytes re-stimulated *in vitro* with PPD. The secretion of IL-2, TNF-α, IFN-γ, IL-17A, and IL-10 was strongly elevated in BCG-vaccinated mice as compared to non-vaccinated mice, and PCI additionally and significantly triggered the release of all cytokines but IL-10 (**Figure 1D**). The beneficial effect of PCI on T-cell responses upon *M. bovis* BCG vaccination was confirmed using three different strains of BCG (**Figure 1E**), i.e., Pasteur *M. bovis* BCG (Past. BCG), Denmark *M. bovis* BCG (DK BCG) as well as a genetically modified Pasteur *M. bovis* BCG lacking a zinc metalloprotease gene (Past. BCG*Δzmp1*) (70).

### Light-Activation of the Photosensitizer Was Indispensable for the Effect of PCI on BCG Vaccination

**Figure 2:**
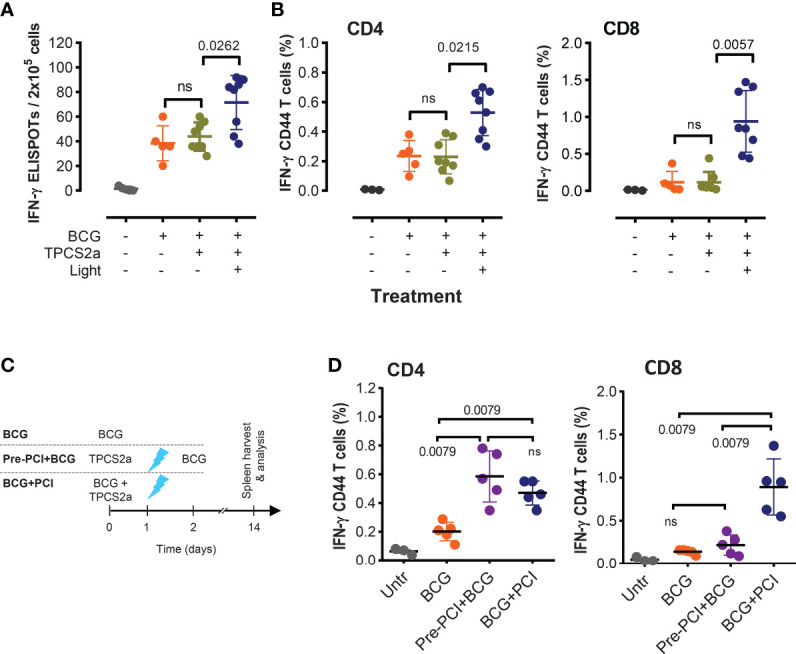
Light is indispensable for the adjuvant effect of PCI in *M. bovis* BCG vaccination. **(A, B)** Groups of mice were vaccinated with 4×10^6^ CFU BCG mixed with TPCS2a (n=16), BCG only (n=5), or left untreated (n=5). Half the mice receiving TPCS2a was light treated after 18 h, while the other half received no light treatment. Two weeks after vaccination, splenocytes were assessed for PPD-specific T-cell responses by IFN-γ ELISPOT **(A)**, or by flow cytometry **(B)** after staining of IFN-γ-producing and CD44-expressing CD4 and CD8 T cells. **(C, D)** Comparison of T-cell responses in mice receiving combined BCG and PCI treatment or time-separated BCG and PCI. As shown in the scheme **(C)**, one group of mice was treated with admixed BCG and TPCS2a, followed by light exposure 18 hours later (BCG+PCI, n=5). Another group of mice received TPCS2a on day 0, light on day 1, and BCG on day 2 (Pre-PCI+BCG, n=5). Two weeks after BCG vaccination, PPD specific T-cell responses were analyzed by staining and flow cytometry **(D)**. Shown are representative means ± SD from one out of three experiments, and data from all BCG-treated groups were analyzed by Kruskal-Wallis tests.

The illumination is expected to play a key role in PCI-mediated delivery of antigens from the phagosomal compartment into the cytosol, where the antigen can be loaded on MHC class I molecules for subsequent cross presentation to T cells. To test the light effect in PCI-based vaccination with *M. bovis* BCG, C57BL/6 mice were vaccinated with BCG and TPCS2a. Half of all animals received light treatment after 18 hours, while the other half was not treated with light. Mice vaccinated with BCG alone or left untreated were included as controls, but also received light treatment as for the PCI-treated group. Two weeks later, PPD specific T-cell activation was detected in all BCG-vaccinated mice (**Figures 2A, B** **)**. Similar levels of IFN-γ produced by CD4 and CD8 T cells was observed when comparing the BCG and the BCG+TPCS2a groups, while significantly more intracellular IFN-γ was observed in CD8 and CD4 T cells from mice vaccinated with BCG+TPCS2a+light (**Figures 1**, **2A, B** **)**. These results suggest that the photosensitizer is not *per se* an adjuvant, but that light is indispensable for the PCI-mediated enhancement of T-cell responses. However, photosensitizer itself did not influence the immunogenicity of *M. bovis* BCG.

### Administration of BCG Together With Photosensitizer Was Essential for Triggering a Strong CD8 T-Cell Activation

**Figure 3:**
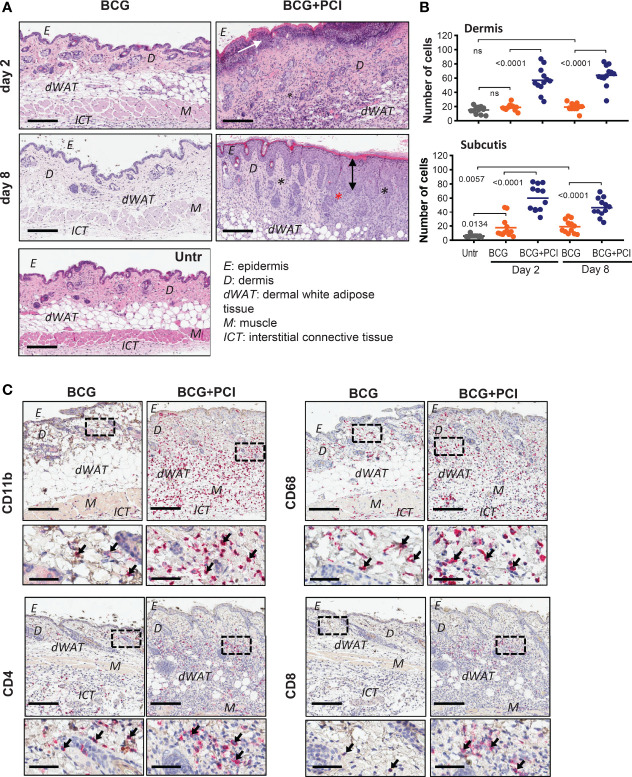
PCI-mediated tissue inflammation in mouse skin. **(A)** H&E staining of belly skin sections harvested 2 or 8 days after *M. bovis* BCG vaccination (BCG) or BCG combined with photosensitizer and light (BCG+PCI). Scale bar: 200 μm. Black asterisks indicate inflammatory infiltrate composed mostly of neutrophils, eosinophils and histiocytes. White arrow points to necrotic epidermis; black arrow indicates acanthosis (thickened epidermis). Representative pictures of two mice per group are shown. **(B)** Quantification of cell numbers in dermis and dermal white adipose tissue (subcutis) from control and treated mice (n=2). Each dot represents number of cells counted in one out of 6-7 frames per section and mouse. The data were analyzed by one-way ANOVA with Bonferroni’s multiple comparison test. **(C)** Immunohistochemistry of skin sections for detection of CD11b, CD68, CD4, and CD4 expression. Scale bar: 200 μm and 50 μm for zoomed-in sections. Arrows indicate stained immune cells.

We next investigated whether the inflammation induced by TPCS2a and light affected subsequent vaccination with *M. bovis* BCG. To this end, one group of mice was photochemically treated prior to the BCG vaccination: TPCS2a on day 0, light on day 1, BCG on day 2. Another group was vaccinated with the mixture of *M. bovis* BCG and TPCS2a on day 0 and received light treatment on day 1 as normal (**Figure 2C**). Two weeks after vaccination, more IFN-γ-producing CD4 T cells were observed in PCI-treated animals than in mice vaccinated with BCG only (p=0.0079), independently of the order of the photochemical treatment and vaccination (**Figure 2D**, left panel). In contrast, when TPCS2a and light were administered prior to BCG vaccination, no added effect on the CD8 T-cell responses was observed when compared to BCG only (**Figure 2D**, right panel). However, highly enhanced CD8 T-cell responses were observed in splenocytes of mice that received parallel BCG and PCI treatment. This result suggests that photochemical treatment causes a general inflammation with adjuvant effect probably involving the recruitment of inflammatory cells, which again aid in the transport mycobacterial antigens to the LNs for classical MHC class-II CD4 T-cell activation (cf. **Figure 3**). This adjuvant effect, however, is probably not including the CD8 arm of the T-cell responses. For this, light has to be given after BCG and photosensitizer, as to enable cytosolic internalization and MHC class-I associated processing of the antigen.

### PCI With *M. bovis* BCG but Not Vaccination Alone Caused Local Inflammation in the Skin

A certain level of inflammation is expected to be beneficial for the induction antigen-specific immune responses. Local skin inflammation was detected in mice that received the BCG vaccine and consecutive PCI treatment, observed as inflammatory infiltrate (composed of neutrophils, eosinophils and histiocytes), thickening of epidermis (acanthosis) and edema. Additionally, activation of a photosensitizer by illumination resulted in ulceration, necrosis of epidermis and compact hyperkeratosis (**Figure 3A**). Formation of fibrotic scar tissue was observed eight days after the BCG+PCI vaccination (data not shown). Cellular infiltrate in dermis and dermal white adipose tissue was significantly increased in the BCG- and PCI-treated mice when compared to the control group receiving BCG only (**Figure 3B**). In addition, immunohistochemistry revealed that PCI increased infiltration of CD11b-positive neutrophils and macrophages, CD68 macrophages, CD4 helper T cells and CD8 cytotoxic T cells to the treated skin. Infiltrate was localized in lower dermis, dermal white adipose tissue and upper interstitial connective tissue (**Figure 3C**). Increased infiltration of F4/80 macrophages and CD11c DCs was not observed (data not shown). For the non-PCI treated mice, the infiltrate was localized in dermis, dermal white adipose tissue, muscle, and in upper interstitial connective tissue.

### Enhanced Epitope-Specific CD8 T-Cell Activation by PCI-Based BCG Vaccination

**Figure 4:**
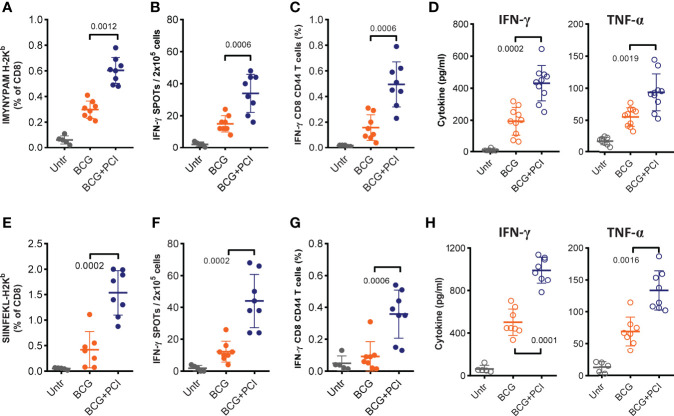
MHC class-I-restricted epitope-specific CD8 T-cell responses measured after PCI-based BCG::OVA vaccination. Mice were spiked with 1×10^6^ naïve OT-I cells one day prior to vaccination with 2×10^6^ CFU of BCG::OVA (BCG) mixed with 50 µg TPCS2a (BCG+PCI, n=8) or BCG (n=8) only. Control mice were left untreated (Untr, n=5). After 18 h, all mice were illuminated for 6 min. Seven days after vaccination, mice were bled and stained with MHC class I-pentamers for IMYNYPAM **(A)** or SIINFEKL **(E)** and analyzed by flow cytometry for the frequency of pentamer- and CD8-positive T cells. Two weeks after vaccination, mice were euthanized, and epitope-specific T-cell responses were assessed in splenocytes either with IFN-γ ELISPOT **(B, F)** or by flow cytometry for assessment of intracellular IFN-γ production in CD8 T cells with CD44 expression **(C, G)**. Splenocytes were also re-stimulated *in vitro* with each peptide for analysis of specific IFN-γ and TNF-α cytokine production in supernatants **(D, H)**. Experiments were repeated three times. The shown data are means ± SD and were analyzed by Mann-Whitney.

CD8 T-cell responses are expected to be important for effective prevention or treatment of TB infection. To evaluate epitope-specific CD8 T-cell responses as a function of PCI, the intrinsic mycobacterial H-2k^b^ MHC class-I-binding epitope IMYNYPAM was used. In addition, the Denmark *M. bovis* BCG strain was genetically modified to express the H-2k^b^ MHC class-I-binding epitope SIINFEKL from ovalbumin (71). To facilitate analysis of the SIINFEKL response, lymphocytes from naïve OT-I transgenic mice were adoptively transferred into recipient C57BL/6 mice one day prior to vaccination. One week after vaccination, venous blood was collected and flow cytometry revealed increased frequencies of IMYNYPAM- (**Figure 4A**) and SIINFEKL- (**Figure 4E**) specific CD8 T cells as compared to blood from untreated animals. The subsequent PCI treatment significantly facilitated CD8 T-cell proliferation for IMYNYPAM (p<0.005) and SIINFEKL (p<0.001). Three weeks after vaccination, splenocyte analysis *in vitro* also demonstrated more robust IMYNYPAM- and SIINFEKL-specific CD8 T-cell responses after PCI treatment and as measured by IFN-γ producing CD8 T cells by ELISPOT (**Figures 4B, F**) and intracellular cytokine staining and flow cytometry (**Figures 4C, G**). Finally, splenocytes from mice vaccinated with BCG and PCI secreted significantly more IFN-γ and TNF-α cytokines into cultures of cells re-stimulated with IMYNYPAM or SIINFEKL (**Figures 4D, H**).

### Comparison of T-Cell Responses Between Intradermal BCG+PCI and Intravenous BCG Vaccination

**Figure 5:**
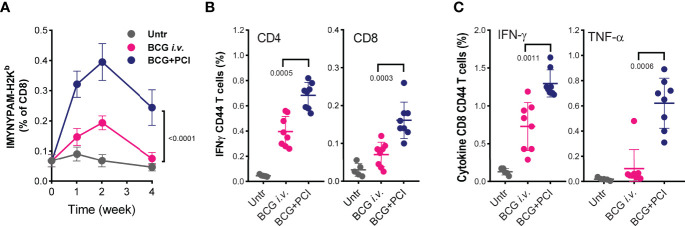
Comparison of T-cell responses after PCI-based BCG vaccination and i.v. BCG vaccination. Mice were vaccinated either with i.v. administration of 4×10^6^ CFU BCG (BCG i.v.; n=8), or with PCI-based BCG vaccination (BCG+PCI; n=8). Untreated mice were used as control (n=5). After vaccination, all mice were bled at weeks 1, 2, and 4, and PBMCs were analyzed for IMYNYPAM-specific CD8 T-cell proliferation *in vivo* using specific pentamer staining and flow cytometry and two-way ANOVA test was applied **(A)**. Nine weeks after vaccination, PPD specific CD4 and CD8 T-cell responses **(B)** and IMYNYPAM specific IFN-γ (**C**, left) and TNF-α (**C**, right) production in CD8 T cells were analyzed in splenocytes by flow cytometry. Means ± SDs are shown, and p-values were analyzed using two-tailed non-parametric Mann Whitney U-tests.

Recently, *i.v.* BCG vaccination was proposed to improve efficacy of BCG vaccination (26). Therefore, we compared T-cell responses induced by PCI-based and *i.v.* BCG vaccination. The frequency of IMYNYPAM-specific CD8 T cells in blood of mice was significantly higher (p<0.0001) after PCI-based BCG vaccination than after *i.v.* BCG administration, and the response was more long lasting (**Figure 5A**). Nine weeks post vaccination, significantly higher frequency of IMYNYPAM-specific IFN-γ producing CD8 T cells were detected in splenocytes (**Figure 5B**), and consistently, more PPD-specific IFN-γ producing CD4 and CD8 T cells and more TNF-α producing CD8 T cells were determined after PCI-based BCG vaccination than that after *i.v.* BCG vaccination (**Figure 5C**).

### PCI Did Not Change the Drainage of BCG to Lymph Nodes, but Facilitated CD8 T-Cell Effector Outcome

In order to track and localize processing and cross-presentation of mycobacterial antigens upon PCI-aided BCG vaccination, groups of mice were vaccinated with BCG::OVA and PCI or with BCG::OVA only. Two days later, LNs and spleens were isolated and used as MHC class I antigen-bearing APCs and co-cultured with naïve H-2b OT-I CD8 T cells. Inguinal, but not mesenteric and axillary LN cells from vaccinated mice triggered IFN-γ production in the co-cultures with OT-I cells (**Supplementary Figure S2**), suggesting that PCI does not change the drainage pattern of BCG and BCG-carrying APCs. However, PCI led to increased secretion of IFN-γ when OT-I cells were co-cultured with splenocytes from BCG-vaccinated mice.

### PCI Treatment Elicited the Activation of BCG-Loaded Macrophages *In Vitro*

**Figure 6:**
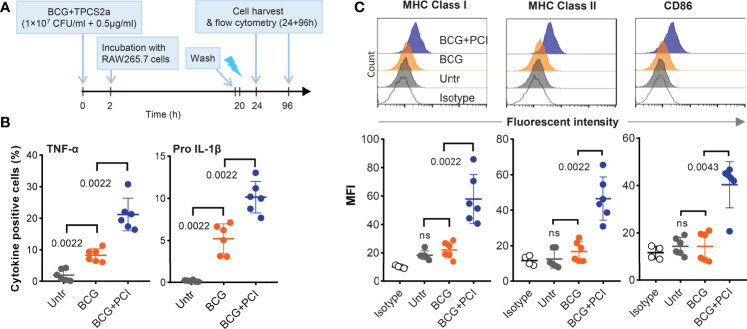
PCI treatment induced macrophage activation *in vitro*. **(A)** Experimental scheme. BCG (1×10^7^ CFU/ml) and TPCS2a (0.5 µg/ml) were mixed and co-incubated at 37°C and for 2 hours in PBS. Control samples contained BCG only. After washing thrice in PBS to remove non-bound photosensitizer, BCG-TPCS2a samples or BCG only were added to RAW 264.7 macrophage cultures (MOI 2:1). Negative control samples contained macrophages only. The samples were incubated overnight at 37°C, washed thrice, and treated with light at 2.43 J/cm^2^. **(B)** Four hours after light exposure, the macrophages were tested for the production of TNF-α and pro IL-1β by flow cytometry. **(C)** Three days after the light treatment, surface expression of MHC-I, MHC-II and CD86 on BCG-PCI loaded macrophages were measured. The histograms are representative of individual treatment and surface expressions are shown as median Fluorescent Intensity. The results summarizes three individual experiments with each two samples and show mean ± SDs. Statistical analysis was done using one-way ANOVA with Bonferroni’s multiple comparison test comparing the indicated groups.

Since macrophages are a major target of *Mtb*, we tested how PCI treatment affected macrophage activation, antigen processing, and presentation upon BCG vaccination. First, BCG were mixed with different concentrations of TPCS2a for 1 or 18 hours, then were seeded on 7H10 bacterial culture petri dishes to check the viability. Such co-incubation of BCG and TPCS2a did not affect the viability of BCG, since BCG bacterial colonies obtained from TPCS2a treated BCG were comparable to those obtained from BCG without treatment in when mycobacterial growth was tested (**Supplementary Figure S3**). To this end, *M. bovis* BCG was incubated with TPCS2a for two hours to allow binding of photosensitizer on BCG. After removal of free TPCS2a, the BCG-TPCS2a combination or BCG alone was incubated with RAW264.7 macrophages overnight to allow BCG uptake (**Figure 6A**). The macrophage cultures were harvested, washed to remove the any free BCG, and illuminated to trigger the activation of BCG-associated TPCS2a. The cells were split in two to (i) test the activation status and the secretion of pro-inflammatory cytokines after further 24 hours culturing or (ii) to use for intralymphatic immunization (see below).

Firstly, TNF-α and pro-IL-1β secreted by BCG-infected and PCI-treated RAW264.7 cells were analyzed by flow cytometry (**Figure 6B**). Compared to untreated RAW264.7 cells, significantly higher frequencies of cytokine-producing cells were measured in BCG-infected RAW264.7 macrophages. Approx. 10% of all cells produced TNF-α and 5% produced pro IL-1β. The corresponding frequencies for BCG-infected and PCI-treated macrophages were approx. 20% and 10%. In addition, a clear upregulation of MHC class I, MHC class II, and co-stimulatory CD86 molecules was observed on macrophages treated with both BCG and PCI, but not on macrophages only infected with BCG or left untreated (**Figure 6C**). These results collectively suggest that PCI treatment optimized the fitness of macrophages for more effective presentation of mycobacterial antigens after BCG infection.

**Figure 7:**
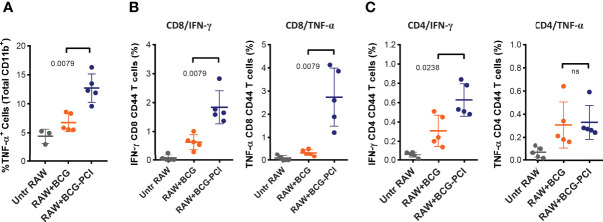
Strong T cell activation induced by the intralymphatic transfer of BCG/PCI-treated RAW264.7 macrophages. Groups of five BALB/c mice were immunized with 1×10^6^ RAW264.7 macrophages loaded with BCG, or BCG and TPCS2a as described in **Figure 6**. The cellular vaccines were injected directly into inguinal LNs. Six days later, the mice were euthanized and inflammatory responses were monitored in one inguinal LN from each animal with intracellular staining of TNF-α producing CD11b^+^ cells **(A)**. The second inguinal LNs were analyzed for PPD-specific IFN-γ and TNF-α production in CD4 **(B)** and CD8 **(C)** T cells. Means ± SDs are shown from one out of two representative experiments, and the data were analyzed by Mann-Whitney.

Secondly, and to test how the improved activation and antigen-presentation status of the macrophages *in vitro* translated into antigen-presentation and immune response elicitation *in vivo*, BCG- and PCI-treated RAW264.7 macrophages were injected directly into the inguinal LNs of syngeneic BALB/c mice. Six days later, the mice were euthanized and the inguinal LNs were removed and analyzed by flow cytometry. Interestingly, TNF-α-producing CD11b-positive cells were detected in all LN preparations, the strongest response observed in animals immunized with BCG- and PCI-treated RAW264.7 macrophages (**Figure 7A**). Hence, the macrophages treated with PCI *in vitro* also induced strongest local inflammation *in vivo*. Next, the activation of CD8 (**Figure 7B**) and CD4 (**Figure 7C**) T cells in the injected LNs was determined. While BCG-infected macrophages only induced IFN-γ production in inguinal CD8 T cells, PCI-treated and BCG-infected macrophages triggered both IFN-γ- and TNF-α-production in inguinal CD8 T cells. The analysis of CD4 cells from mice immunized with macrophages revealed beneficial effect of PCI for IFN-γ production but not for TNF-α- production.

## Discussion

Numerous vaccines have been investigated for their efficacy in preventing TB. While viral or nucleic acid vaccines (76–79) deliver genetic material into the host APCs, potentially resulting in endogenous transcription and subsequent translation into proteins and therefore effective presentation on MHC class I, protein vaccines or whole-cell bacterial vaccines usually do not gain cytosolic access. Adjuvants promoting CD8-mediated immunity are therefore a key element for developing effective subunit TB vaccines (80, 81). Many adjuvants such as aluminum salts, TLR ligands, or synthetic particles may strengthen immunogenicity, albeit cross-presentation of CD8 T-cell epitopes may still be ineffective. In this study, we demonstrated that *M. bovis* BCG could be combined with a photochemical compounds that upon light activation causes leakage of phagosomes with cytosolic release of antigen for cross-presentation of antigen to CD8 T cells. This so-called photochemical internalization (PCI) may represent a new approach for targeting of CD8 T cells with TB vaccines.

The objective of the current study was dual. Firstly, we tested the possibility of using PCI for targeting of a live bacterial vector to the cytosol of APCs, as to trigger MHC class-I-mediated CD8 T-cell responses. The targeting vehicle was the photosensitizer TPCS2a, which through light activation should mediate phagosomal or phagolysosomal leakage, causing the cytosolic release of the bacterium, or protein digests thereof. As a bacterial vector, we used *M. bo*vis BCG, an almost 100 years old vaccine against *M. tuberculosis*. *M. bo*vis BCG is on the World Health Organization’s list of essential medicines, but has been taken off many national immunization programs in industrialized countries, due to low protective efficacy in pulmonary TB in adults (82). In part, this inefficacy has been ascribed to immunological evasion mechanisms that arrest the bacteria within the phagosomes and hamper bacterial transport to draining lymph nodes (83). Hence, the processing and presentation of mycobacterial antigens are impaired (84). For *Mtb*, the same mechanism allows sufficient time to establish an infection (85, 86). This leads us to the second objective of the current study, which was to test if the impaired T-cell responses of *M. bovis* BCG could be improved by applying PCI to the vaccination procedure. To this end, mice were vaccinated by intradermal injection of *M. bovis* BCG, with or without the photosensitizer TPCS2a.

The expected series of events after vaccination with *M. bovis* BCG is that the inoculum causes a local inflammation in the skin by triggering release of inflammatory molecules from keratinocytes and other resident dermal cells. The chemo-attractive molecules lead to infiltration of other immune cells, especially neutrophils, but also professional APCs such as macrophages, which would possibly take up the bacteria by phagocytosis. It is well know that macrophages and other professional APCs may migrate to draining lymph nodes for presentation of antigens to lymphocytes and for stimulation of adaptive immune responses (87). More recently, it has also been suggested that neutrophils can also engage in antigen presentation, by draining to lymph nodes (88–90). The current study demonstrated that PCI strongly improved antigen-specific T-cell responses after vaccination with *M. bovis* BCG. Especially, CD8 T-cell responses were enhanced. One reason for the increased immunogenicity may be due to the increased overall inflammation in the skin upon PCI treatment as demonstrated by the histological analysis of the mouse skin. The higher number of infiltrating immune cells in skin of PCI-treated mice naturally increased the probability of APCs taking up bacteria and presenting antigen to lymphocytes in the lymph nodes. By the same token, it has been suggested that scar formation after BCG vaccination may be associated with and a surrogate marker for better T-cell responses (91), protection (92), and survival (93). One can therefore speculate that the PCI-mediated inflammation or even a certain level of scaring or ulceration may cause scare-like reactions that facilitates the anti-mycobacterial immune responses and protection. Of note, very high photosensitizer or light doses may cause excess inflammation and even necrosis, which of course could be detrimental for the subsequent stimulation of adoptive immune responses, and inacceptable in prophylactic vaccines. Correspondingly, the level of inflammation or vaccine reactogenicity upon PCI-based vaccination can be regulated adjusting the photosensitizer or light dose. However, and most interestingly, the data suggest that not inflammation alone leads to the increased CD8 T-cell responses. When skin inflammation was induced 1-2 days prior to vaccination, only CD4 T cells, but not CD8 T cells were activated. Here, we may speculate that the APCs migrated out of the skin before the vaccine was applied. Hence, remnant APCs taking up the vaccine could not be reached by light. In contrast, the increased frequencies of observed CD8 T cells after PCI-mediated vaccination suggest that light activation of TPCS2a resulted in antigen release from phagosomes into the cytosol. In this situation, the antigen can directly access the MHC I pathway of antigen processing and presentation and cross-present short epitopes to CD8 T cells, while non-released antigen would remain in the phago/lysosomes for subsequent presentation *via* MHC class II. This relocation from default MHC class II to MHC class I antigen presentation was strictly dependent on light, because mice vaccinated with a combination of *M. bovis* BCG and TPCS2a did not improve CD8 T-cell responses when light was not applied. Although we have previously shown this phenomenon for soluble proteins (59–61, 65, 66) as well as for synthetic vaccine particles (62–64), our current study is the first demonstration that a live bacterial vector could be combined with PCI for cytosolic antigen delivery and for stimulation of CD8 T-cell responses.

The stimulation of CD8 T-cell responses against *M. bovis* BCG have been the objective in much TB vaccine research and development (25–27, 94). Having a large genome and being relatively easy to manipulate, a great variety of recombinant variants of *M. bovis* BCG have been studied and tested pre-clinically [see review (29)]. Many of these vaccine candidates express immune modulatory compounds such as cytokines IL-2, IL-18, GM-CSF, or IFN-γ (47, 95–97). Other overexpress mycobacterial antigens against which T-cell responses have proven protective potential or the BCG had deletions in genes supposed to be involved in the phagosomal arrest and reduced T-cell responses (20–23, 36, 70). It has been reported that VPM1002 (BCG *ΔureC*:hly) expresses the *Listeria monocytogenes* protein listeriolysin O, a protein that causes lysis of the phagosome and thereby cytosolic release of BCG for stimulation of CD8 T-cell responses and is currently tested in phase III trials to study efficacy in prevention of TB recurrence in adults (NCT03152903) and for prevention of *Mtb* infection in infants (NCT04351685) (36). Further vaccine candidates with potential CD8 T-cell stimulating properties are live, attenuated, non-replicating viruses, such as adenovirus and the attenuated poxvirus modified vaccinia virus Ankara (MVA) expressing mycobacterial and adjuvant protein (98). However, a possible problem with viral vectors in vaccines is the induction of vector-specific antibodies that can inhibit effect of later booster vaccinations. While one phase IIb trial with MVA85A showed higher frequencies of BCG-specific IFN-γ-secreting cells, as quantified by ELISPOT assay, another phase IIb trial could not confirm protection (99). However, it is generally recognized that the induction of IFN-γ-secreting T cells are associated with a reduced risk of developing TB (37), again underlying the important of T-cell responses in TB.

In our study, BCG-specific IFN-γ-secreting cells could be measured, by ELISPOT, ELISA, and by flow cytometry, and the absolute number as well as frequency of such cells were significantly increasing in mice that received PCI treatment with the vaccination. The T-cell responses induced by PCI-mediated BCG vaccination was typically diverse with the T cells producing IFN-γ, TNF-α, IL-2, and IL-17. The latter results aligns well with published reports suggesting that Th17 cells, which naturally traffic to the airways, can accelerate the recruitment of protective Th1 cells in pulmonary TB (100–102). Indeed, rhesus macaques were protected against a Mtb challenge when vaccination induced pulmonary Th1 and Th17 cells that also expressed IFN-γ, TNF-α, IL-2 and IL-17 (103). Such multifunctional T cells play vital roles in the prevention of Mtb infection (104) protection depending mainly on the IFN-γ- and IL-2-expressing Th1 cells. IFN-γ is a key effector cytokine in the control of Mtb infection, while IL-2 is a T-cell growth factor that also assures long-term survival of lymphocytes (105, 106). IFN-γ-producing cytotoxic CD8 T cells have also been proven vital in the elimination of intracellular bacterial infections including Mtb (107–109), for which reason several Mtb vaccine developments aimed at stimulating also antigen-specific CD8 T cell (110–112).

The administration routes of BCG vaccines have been suggested to be important for vaccine efficacy (52, 113). A recent study demonstrated that intravenous (*i.v.*) administration of BCG to non-human primates prevented TB infection (26). Antigen-responsive CD4 and CD8 T-cell responses in secondary lymphoid organs and in lung tissues were substantially higher after *i.v.* BCG vaccination, compared to vaccination *via* the intradermal route or aerosol delivery. These results confirm that optimal T-cell activation after BCG vaccination may play a key role for prevention of TB infection. In the current study, we also compared the T-cell activation after PCI-mediated intradermal BCG vaccination with *i.v. M. bovis* BCG. While latter produced a robust T-cell response, especially in CD4 T cells, PCI-mediated vaccination again produced relatively stronger CD4 and CD8 T-cell responses.

While previous studies of PCI-based immunization focused on the delivery of soluble protein vaccines, the current study demonstrated that PCI could also be applied to live bacterial vaccine particles for targeted delivery of vaccine antigens to APCs and for cross-presentation of antigens to CD8 T cells. The stimulation of pluripotent and cytotoxic CD8 T cell is important for the prevention of intracellular pathogens such as *M. tuberculosis*, but also for the therapeutic treatment of such pathogens. Similarly, the treatment of chronic infections caused by non-cytopathic viruses such as hepatitis C virus, herpes virus, cytomegalovirus human papillomavirus, human T-cell leukemia viruses, or also HIV, may once be feasible through vaccination, and also here, PCI may pave the way for new immunotherapeutic treatment concepts. Indeed, the first clinical trial testing PCI-based vaccination against human papilloma virus (HPV) was just recently published (114). Next to demonstrating safety and tolerability, the results of this phase I study in more than 90 healthy volunteers confirmed that PCI could facilitate CD4 and CD8 T-cell responses for protein antigens. The combination of PCI with *M. bovis* BCG in humans is still to be tested. Though it is unlikely that a PCI-based TB vaccine will be chosen in worldwide preventive immunization programs, it could find therapeutic application for the treatment of existing infections. Moreover, the concept of cytosolic targeting and the pre-clinical effects may guide further research and development for better vaccines against intracellular pathogens and as well as cancer.

## Data Availability Statement

The raw data supporting the conclusions of this article will be made available by the authors, without undue reservation.

## Ethics Statement

The animal study was reviewed and approved by Kantonale Veterinäramt Zürich.

## Author Contributions

YW-M designed and performed experiments, analyzed data and wrote the manuscript. ZK performed experiments and wrote the manuscript. IK analyzed data. GB, AD, EV, BA, and MM performed experiments. TK and CH provided intellectual input. PS designed experiments and provided intellectual input. PJ designed experiments, performed data analysis and wrote the manuscript. All authors contributed to the article and approved the submitted version.

## Funding

The project has received financial support from *Swiss Cancer League* (KFS-3451-08-2014), Swiss National Science Foundation (CR3313_162773; 310030_197699), Novartis Foundation for Medical-Biological Research, the Norwegian Research Council (Project no. 269817), Truus and Gerrit van Riemsdijk Foundation Vaduz, Swiss State Secretary for Education, Research and Innovation (SERI; #15,0033/643381/1131-52107) based on association with the EU-Funded TBVAC2020 Consortium (#643381) within the Horizon2020 Framework and PCI Biotech.

## Conflict of Interest

AH is employee of PCI Biotech, which has filed patents on the use of photosensitizer in vaccination. AH also owns shares in PCI Biotech. AH and PJ are mentioned as inventors of patents describing the use of PCI in immunization and vaccination.

The remaining author declares that the research was conducted in the absence of any commercial or financial relationships that could be construed as a potential conflict of interest.

This study received funding from PCI Biotech. The funder, represented by co-author AH was not involved in the study design, collection, analysis, and interpretation of data. However, the funder, represented by co-author AH, reviewed that manuscript prior to submission.

## Publisher’s Note

All claims expressed in this article are solely those of the authors and do not necessarily represent those of their affiliated organizations, or those of the publisher, the editors and the reviewers. Any product that may be evaluated in this article, or claim that may be made by its manufacturer, is not guaranteed or endorsed by the publisher.
