# Supplementary material for: Photochemically-Mediated Inflammation and Cross-Presentation of Mycobacterium bovis BCG Proteins Stimulates Strong CD4 and CD8 T-Cell Responses in Mice
Source: Front Immunol. 2022 Jan 31;13:815609. doi: 10.3389/fimmu.2022.815609 (PMC8841863; doi:10.3389/fimmu.2022.815609)
Supplement: Supplementary file 1 [file DataSheet_1.pdf]

Supplementary Materials

Waeckerle-Men, et al.

Photochemically-mediated inflammation and cross-presentation of Mycobacterium bovis BCG proteins stimulates strong CD4 and CD8 T-cell responses in mice

Submission to *Frontiers in Immunology*

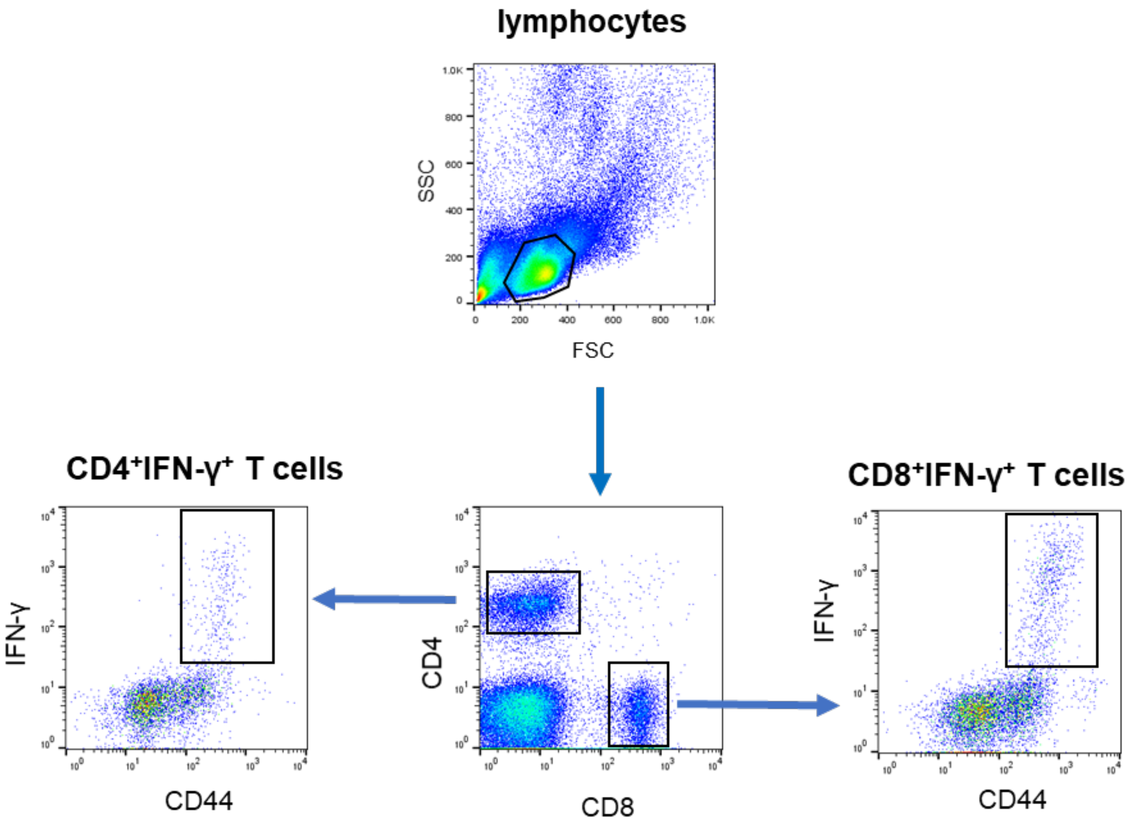

Supplemental Figure S1.

Representative flow cytometry gating strategy for detection of IFN-γ producing CD4 and CD8 T cells in splenocytes of B6 mice after PCI-based BCG vaccination as presented in **Fig. 1C**.

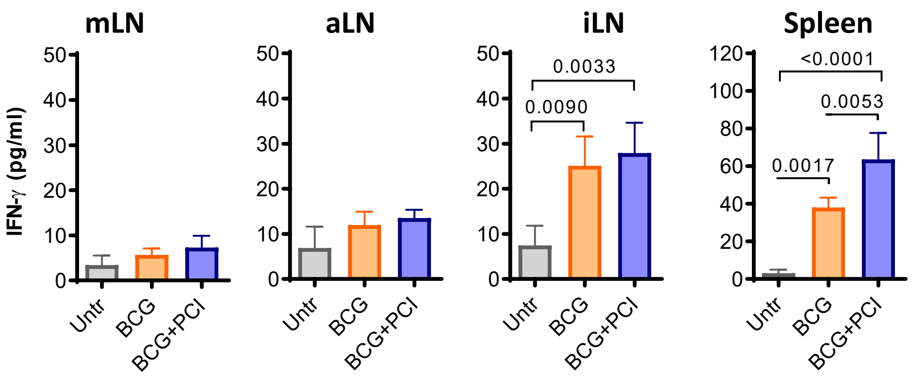

Supplemental Figure S2.

PCI treatment increased MHC class I-restricted antigen-presentation in secondary lymphoid organs after BCG vaccination. Groups of four C57BL/6 mice were *i.d.* vaccinated with BCG::OVA ± PCI and light-treated one day later. Untreated animals were used as control (Untr, n=3). Two days after vaccination, mice were euthanized and spleen, inguinal, mesenteric, and axillary LNs were isolated and single-cell suspension thereof were used as APCs (1×10^5) to present SIINFEKL OT-I cells (2×10^4) in co-cultures. After 72 h, IFN-γ concentrations in the supernatants were measured with ELISA. Means and SDs are shown.

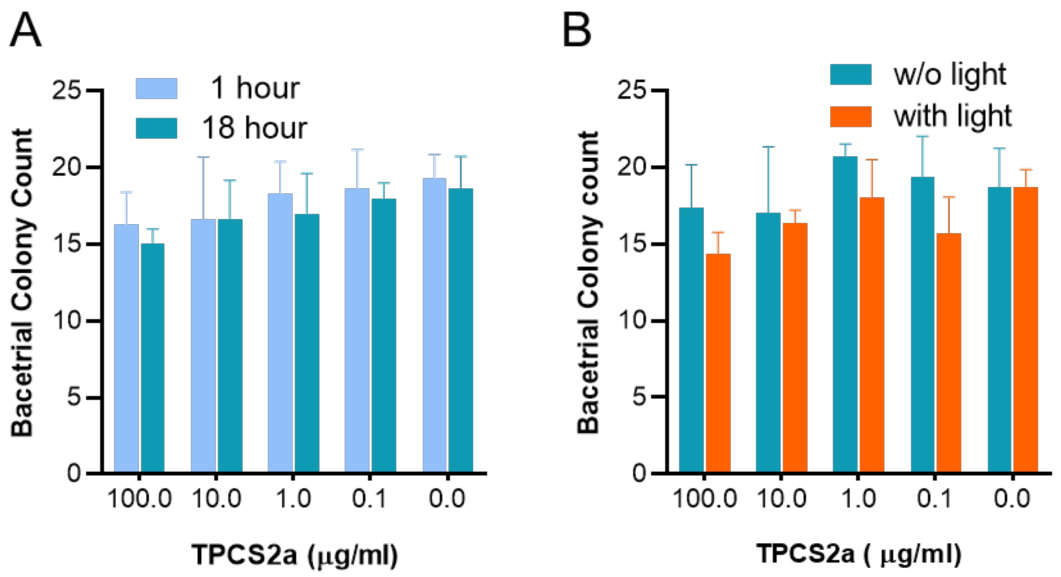

Supplemental Figure S3.

Co-incubation of Photosensitizer TPCS2a and BCG does not affect BCG viability. (**A**) BCG mycobacteria ($2x10^2$ /ml) were incubated with a serial concentration of PS TPCS2a for 1 or 18 hours at 37°C in dark. Mixtures of BCG and TPCS2a were then seeded to 7H10 bacterial culture petri dishes as 100 µl/area, protected from the light, and were incubated at 37°C. After 3 weeks, BCG bacterial colonies were counted and compared with those obtained from BCG without PS (TPCS2a 0.0 µg/ml). Heat-killed (100 °C, 2h) BCG samples were used as negative controls for BCG viability (mean bacterial count < 1; data not shown). (**B**) BCG and TPCS2a were first prepared and incubated for 18 hours as indicated in (**A**). After washed thrice with PBS, half of the mixtures were illuminated for 3 minutes and the other half was left without light treatment. The BCG was then seeded to 7H10 dishes for 3 weeks for mycobacterial colony enumeration. Results are from triplicate BCG cultures from each experiment. Shown are means and SEM from individual groups. The experiments were repeated 2 times with comparable results.
